# Mechanical Plugging Strength and Failure Risk of Low-Melting-Point Alloy in Casing Sealing

**DOI:** 10.3390/ma19071279

**Published:** 2026-03-24

**Authors:** Wenchao Tao, Gonghui Liu, Chunqing Zha, Wei Wang, Wei Liu, Jun Li

**Affiliations:** 1 College of Mechanical and Energy Engineering, Beijing University of Technology, Beijing 100124, China; twc18332179810@emails.bjut.edu.cn (W.T.); lgh_1029@163.com (G.L.); wangwei53_bjut@163.com (W.W.); 2 CNPC Engineering Technology R&D Co., Ltd., Beijing 102206, China; liuweidri@cnpc.com.cn; 3 College of Petroleum Engineering, China University of Petroleum Beijing, Beijing 102249, China; lijun446@vip.163.com

**Keywords:** low-melting-point alloy, casing sealing, overlying axial pressure, ultimate shear strength, sealing integrity

## Abstract

**Highlights:**

- Based on the maximum shear strength theory, experiments show that the ultimate shear strength between low-melting-point alloy (LMPA) plug and casing decreases linearly with rising temperature. Temperature and axial pressure are identified as key external loads governing LMPA plugging performance, supporting field adaptability evaluation.
- Overlying axial pressure promotes plug expansion and enhances sealing integrity.
- The aspect ratio (*L/D*) significantly affects post-failure tightness. A critical *L/D* ≥ 3.5 is proposed for emergency sealing, offering direct guidance for plugging device structural optimization.

**Abstract:**

To investigate the effects of overlying axial pressure and ambient temperature on the mechanical plugging performance of low-melting-point alloy (LMPA) in casing sealing, simulation experiments were conducted with LMPA as the sealing material to clarify the underlying mechanisms. Based on the maximum shear strength theory of the alloy plug/casing interface, a metal plug forming device and a gas-tightness detection apparatus were designed. Laboratory tests were then carried out to evaluate the ultimate shear strength and gas sealing integrity of the alloy plug, followed by the analysis of forming axial pressure and ambient temperature on mechanical sealing behavior and the exploration of alloy plug failure modes under mechanical plugging conditions. Experimental results show that the ultimate shear strength between the alloy plug and casing decreases with increasing ambient temperature at an average rate of ~0.225 MPa/°C. Applying forming pressure can enhance the integrity of the alloy plug, promote its radial expansion, and thus improve sealing integrity. After sliding shear failure, alloy plugs with an aspect ratio of 2.32 lose gas tightness completely, while those with an aspect ratio ≥ 3.5 retain more than 50% of their original gas sealing capacity after sliding shear displacement. This study provides a theoretical basis and technical reference for the application of LMPA in downhole casing plugging operations.

## 1. Introduction

Carbon capture, utilization, and storage (CCUS) are recognized as one of the most promising technologies to mitigate carbon emissions in the petroleum industry. As a critical component of CCUS technology, the secure storage of CO_2_ in depleted oil and gas reservoirs is essential for ensuring the long-term effectiveness of carbon emission reduction. However, abandoned wellbores can create connecting pathways between oil-, gas-, or brine-bearing formations and groundwater or seawater systems, thereby posing significant risks of contamination to freshwater aquifers and marine environments [1]. This risk highlights the importance of reliable wellbore isolation, which is a common essential requirement for both CCUS operations and well plugging and abandonment (P&A). Specifically, both technologies demand the establishment of a secure and durable barrier within the wellbore to prevent fluid migration.

Currently, cement-based materials are the predominant choice for wellbore sealing in CCUS and P&A operations worldwide, as they have been widely utilized for decades due to their availability and cost-effectiveness. However, in most cases, placing a cement plug in a cased wellbore is inadequate to prevent gas migration [2,3]. Downhole complexities, including hydration shrinkage, mechanical loading, thermal stresses, and corrosive downhole environments, can compromise cement integrity, thereby potentially leading to cracking or debonding of the cement plug and the subsequent formation of leakage pathways [4,5,6]. Once such pathways are formed, significant environmental contamination (e.g., groundwater pollution) and substantial economic losses will occur, which further exacerbates the challenges associated with CCUS and P&A operations. Consequently, in recent years, researchers have focused growing attention on exploring sustainable alternative materials to replace cement for wellbore sealing. Developing such materials offers significant potential for reducing operational costs in CCUS and P&A projects while supporting environmental preservation, particularly in addressing the inherent limitations of cement-based sealing materials.

Low-melting-point alloys (LMPAs), primarily composed of bismuth, exhibit slight volumetric expansion upon solidification, thereby enabling them to form a tight bond with casing walls [7,8,9]. Additionally, bismuth-based LMPAs possess high density, excellent fluidity in the molten state, and excellent resistance to corrosive downhole fluids such as H_2_S and CO_2_ under downhole conditions [10]. These advantageous properties make bismuth-based LMPAs promising candidate materials for downhole casing plugs, addressing the inherent limitations of conventional cement-based sealing materials. In recent years, an increasing number of researchers and institutions have proposed the application of LMPAs as casing barrier materials. For instance, Zhang et al. [8,9] examined the wettability and bonding strength between LMPAs and various rock types, and analyzed the interfacial adhesion mechanism under different lithological conditions. Field applications have further validated the effectiveness of LMPAs in well abandonment and sealing operations, demonstrating their capacity for efficient and high-quality wellbore plugging [11,12]. In comparative studies, Lewaa et al. [13,14] investigated the shear-bond strength and gas tightness of bismuth-tin (BiSn) alloy plugs compared with cement plugs prepared under identical conditions. Their results confirmed that bismuth-based alloy plugs exhibit significantly superior sealing integrity compared with conventional cement plugs. Furthermore, their work highlighted the role of applied axial forming pressure in enhancing the interfacial bonding between the alloy plug and the casing. To further elucidate the influence of overburden forming axial pressure and ambient temperature on the mechanical sealing integrity of LMPA plugs, this study conducted laboratory simulated experiments to explore the underlying mechanisms governing the mechanical sealing behavior.

To investigate the influence of overlying axial pressure and ambient temperature on the sealing integrity of LMPA plugs in casing applications, this study designed and built experimental apparatus for plug formation and gas tightness testing. This paper presents experimental investigations into the ultimate shear strength and gas sealing integrity of the interfacial bond between alloy plugs and casing. Microstructural analysis of the alloy-casing sealing interface was conducted via optical microscopy to reveal the bonding mechanism. Additionally, the effects of overlying axial pressure and ambient temperature on the sealing performance of the alloy/casing system are systematically examined, and the mechanical failure modes of alloy plugs under downhole sealing conditions are identified. The results obtained provide theoretical support for the development of LMPAS as a novel casing plugging material for downhole wellbore plugging applications. This material is expected to overcome the inherent drawbacks of conventional cement-based plugs and facilitate the engineering application of LMPA plugs in CCUS and plug-and-abandonment (P&A) operations.

## 2. Materials and Methods

### 2.1. Materials

To identify suitable wellbore plugging materials, BiSn alloy samples with varying compositions were prepared by mixing bismuth (Bi) and tin (Sn) in different mass ratios. The materials evaluated included Sn-xBi alloys (where x represents the mass fraction of Bi) with bismuth contents of x = 20, 40, 58 (eutectic composition), and 80, as well as pure bismuth (Bi) and pure tin (Sn) samples. The microstructural and phase characteristics of these alloys were examined using scanning electron microscopy (SEM, Quanta 650, Thermo Fisher Scientific, Hillsboro, OR, USA) to screen an appropriate bismuth-based alloy for downhole casing sealing applications.

**Figure 1:**
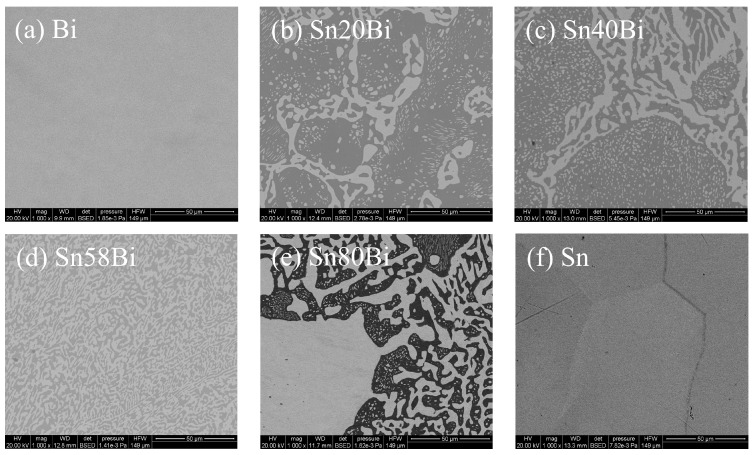
SEM micrographs of SnBi alloys with different mass ratios and the binary phase diagram of the Sn-Bi system [15].

The binary phase diagram and corresponding microstructures of the different Sn-xBi alloys are shown in Figure 1a–f. Here, Figure 1d corresponds to the eutectic composition, while Figure 1c,e correspond to hypoeutectic and hypereutectic compositions, respectively. The alloy samples were prepared by heating mixtures of pure bismuth (Bi) and pure tin (Sn) particles (purity > 99.99%) to 300 °C until fully molten. Prior to melting, 0.5 wt.% of antioxidant flux was added to the metal particle mixtures to prevent oxidation during the melting process. The molten alloy was stirred at a speed of 300 rpm for 10 min to ensure compositional homogeneity and then cooled naturally in ambient air to room temperature. After cooling, the alloy ingots were ground and polished to remove surface oxides and defects for subsequent microstructural characterization.

Microstructural analysis of the BiSn alloys with different bismuth mass fractions revealed that the Sn58Bi eutectic alloy (58 wt.% Bi, 42 wt.% Sn) exhibits superior compositional and microstructural homogeneity, which contributes to enhanced mechanical properties relevant to sealing performance (e.g., ultimate shear strength and hardness). Moreover, as a eutectic alloy, Sn58Bi (a type of LMPA) exhibits a narrow melting range, making it particularly suitable for rapid melting and in situ placement in downhole casing during plugging operations. Consequently, based on the comprehensive evaluation of microstructural homogeneity, melting characteristics, and mechanical properties, the Sn58Bi alloy was selected as the downhole casing plug material for this study. Table 1 lists the key physical properties of the Sn58Bi eutectic alloy.

The casing material used in this experiment is N80 carbon-manganese steel, the surface roughness of the material is Ra 12.5, the inner diameter (ID) of the casing is 21.59 mm, and the outer diameter (OD) is 45 mm. The relevant material properties are shown in Table 2.

### 2.2. Casing Plugging Method with LMPAs

**Figure 2:**
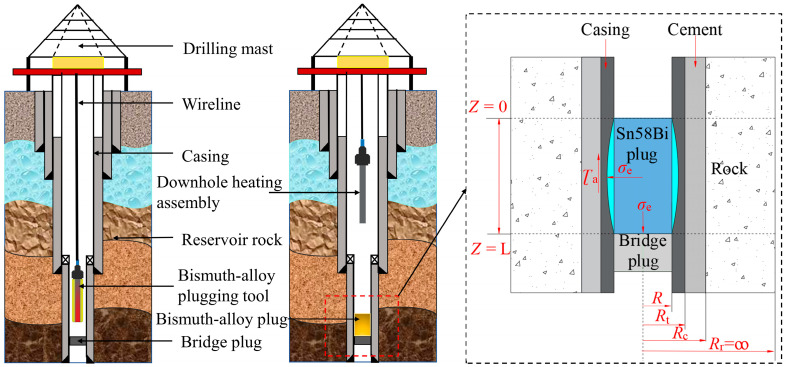
Schematic of wellbore plugging process with LMPA.

When deploying LMPAs for wellbore plugging, the alloy plugging tool was typically lowered to the target isolation interval via a surface rig and wireline unit, using an armored cable for load-bearing and signal transmission. Once the plugging tool was positioned, a heating mechanism—either electrical or thermite-based chemical heating—was activated to melt the LMPA. Currently, thermite-based chemical heating serves as one of the primary methods for this application [18,19,20]. Owing to the high density of the molten LMPA, the molten alloy flows by gravity to fill the casing annulus. As soon as the alloy remains in a liquid state, the heating tool was retrieved to separate the downhole heating assembly from the molten LMPA. After a prescribed cooling period under ambient downhole temperature, the plugging tool was completely withdrawn, and the tool string was inspected for residual alloy contamination. The molten alloy solidifies through heat exchange with the surrounding casing wall and downhole formation, during which it undergoes a slight radial expansion of 0.5–1.0%. This solidification and radial expansion process results in a tight metal-to-metal mechanical seal between the alloy plug and the casing, with sealing integrity maintained primarily through interfacial friction and radial expansion stress. As illustrated in Figure 2, the LMPA plugging tool is deployed by the surface derrick into the plugging interval (above the bridge plug) via armored cable. Upon heating and melting the LMPA coated on the tool, the alloy solidifies above the bridge plug to form a rigid alloy plug, which establishes a reliable metal-to-metal mechanical seal via expansion during solidification. A mechanical bridge plug was set below the target interval—generally within 1.0 m of the target interval—to prevent the downward migration of the molten LMPA into the lower wellbore section.

## 3. Experiments and Processes

### 3.1. Forming Tests of Alloy Plugs

**Figure 3:**
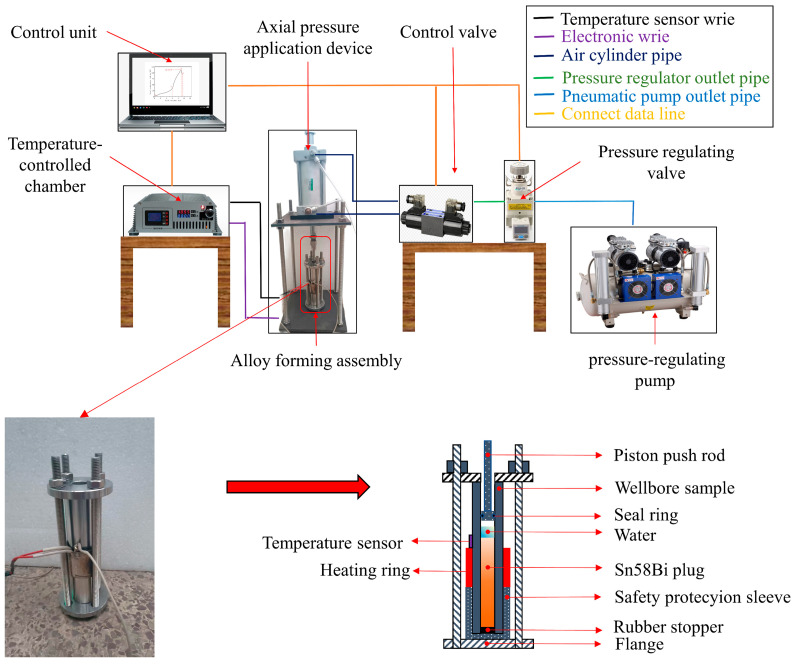
The system of the alloy plug forming device.

To investigate the influence of overlying axial pressure on the forming integrity of alloy plugs, an experimental system was designed to simulate the plug formation process under the combined action of downhole liquid environment and overlying axial pressure. The alloy plug forming test system, as shown in Figure 3, consists of the following components: a control unit, a temperature-controlled chamber (temperature range: 0–500 °C, control accuracy: ±1 °C), an axial pressure application device (pressure range: 0–7.5 MPa, control accuracy: ±0.01 MPa), an alloy plug forming assembly, control valves, a pressure-regulating valve (pressure range: 0–1 MPa), and an air compressor (pressure range: 0–0.8 MPa). The control unit is responsible for coordinating the operation of all components, while the temperature-controlled chamber provides a stable heating environment for alloy melting, and the axial stress application device applies precise axial stress to simulate overburden pressure.

The working principle of the system is as follows: First, the alloy plug forming assembly was pre-assembled, and a wellbore sample (casing material: N80 steel, inner diameter: 21.59 mm, length: 200 mm) was placed inside the assembly. Subsequently, the selected Sn58Bi eutectic alloy and simulated formation water were added to the forming assembly, and the entire test setup was sealed to ensure no fluid leakage. Following the completion of test setup assembly, a specified axial pressure was applied to the top of the alloy plug via the axial pressure application device. The magnitude of the axial stress was controlled by the pressure-regulating valve, which was supplied with a pressure source by the air compressor. Heating was provided by a built-in heating coil in the temperature-controlled chamber, which was regulated by the control unit to melt the Sn58Bi alloy within the wellbore sample. The entire experimental system was designed to simulate the formation process of an alloy plug under a defined overburden axial stress in a simulated downhole liquid environment.

**Figure 4:**
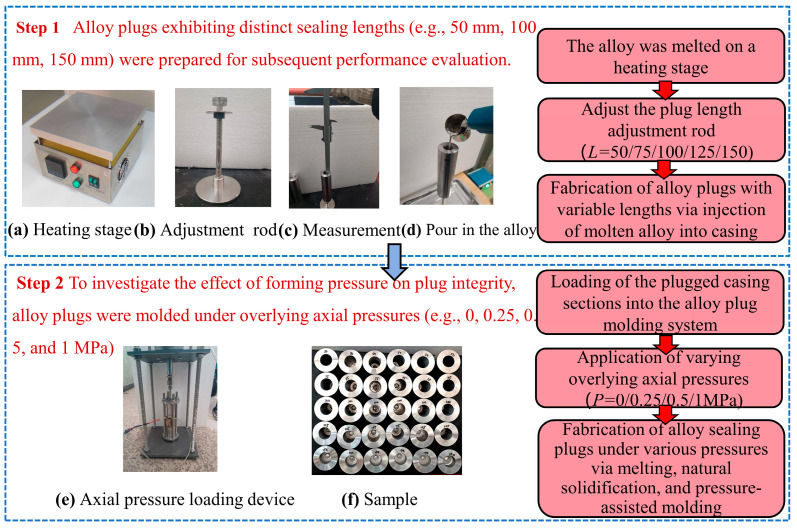
Forming test process of alloy plugs.

In this experiment, alloy plugs with varying sealing lengths (*L* = 50, 75, 100, 125, and 150 mm) were formed inside N80 casing segments (inner diameter: 21.59 mm, outer diameter: 45 mm, length: 200 mm). All plugs were molded under a defined overlying axial pressure. The procedure for the Sn58Bi eutectic alloy plug molding test is shown in Figure 4 and detailed as follows:

(a) Sn58Bi eutectic alloy plugs with different sealing lengths were pre-fabricated inside the N80 casing segments.
(b) The casing segments containing the pre-fabricated alloy plugs were placed into the alloy plug molding assembly and secured tightly to prevent displacement or eccentricity during the molding process.
(c) A 10 mm water column of simulated formation water was added above the alloy plug to simulate the downhole wet environment encountered in actual plugging operations.
(d) The axial pressure application device was activated to apply a specified overlying axial pressure to the top of the alloy plug.
(e) The built-in electric heating system of the temperature-controlled chamber was activated to heat the Sn58Bi alloy at a constant heating rate of 8 °C/min to 200 °C, and this temperature was maintained for 3 h to ensure complete melting and homogeneous distribution of the alloy within the casing segment.
(f) After the sample was cooled naturally to room temperature (20 °C, cooling duration: approximately 2 h), the casing sample with the solidified Sn58Bi alloy plug was carefully removed from the alloy plug molding assembly.

### 3.2. Push-Out Tests of Alloy Plugs

To determine the ultimate shear force required to induce sliding failure of Sn58Bi alloy plugs inside N80 casing segments, a universal testing machine was utilized. In this setup, a hydraulic-driven push rod (diameter: 21 mm) applies an axial force to the end face of the Sn58Bi alloy plug (diameter: 21.59 mm), ensuring concentric alignment between the push rod and the alloy plug to uniformly distribute the axial force, thereby shearing the interfacial bond between the alloy plug and the casing wall and causing relative sliding between the two components. The peak pressure at the initiation of plug sliding by testing specimens fabricated under different overlying axial stresses and test ambient temperatures (30–90 °C) were measured. This enabled the determination of the ultimate shear capacity of the alloy plugs under various influencing factors, which provides a critical basis for evaluating the long-term service performance of Sn58Bi alloy plugs in downhole casing plugging applications. The Sn58Bi alloy exhibits expansion following solidification, which generates radial forces. An optimal curing duration of 24 h is recommended [13]. Accordingly, the magnitude of the expansion force is dependent on the curing time of the Sn58Bi alloy plugs. In this study, all alloy plugs were cured at a specified ambient temperature for at least 24 h to ensure uniform testing conditions when determining their ultimate shear strength.

**Figure 5:**
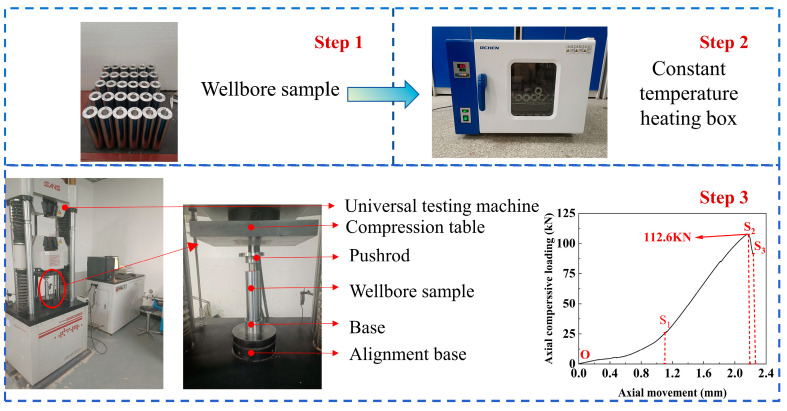
Push-out tests of the LMPA plugs.

The detailed experimental procedure is shown in Figure 5 and outlined as follows:

(a) Sn58Bi alloy plug specimens cured at a constant temperature of 30 °C for 24 h in a constant-temperature incubator were removed and divided into five groups. Each group was then placed inside a constant-temperature heating chamber to preheat to the target test temperature.
(b) The chamber temperature was set to 30 °C, 45 °C, 60 °C, 75 °C, and 90 °C for the five groups, respectively. The specimens were heated at a rate of 5 °C/min and maintained at these set temperatures for 5 h to ensure uniform heating (until the core temperature of the specimens reached the set temperature).
(c) Specimens from each temperature group were quickly transferred within 30 s to the universal testing machine, and the test was initiated within 1 min of transfer to avoid temperature deviation.
(d) The upper compression platen was moved toward the push rod at a speed of 1 mm/min and stopped with a 0.1 mm gap from the rod end, ensuring concentric alignment between the platen, push rod, and alloy plug to avoid eccentric loading.
(e) The testing machine was set to a crosshead speed of 2 mm/min and started; the axial force and displacement data were recorded in real time by the machine’s data acquisition system at a sampling frequency of 10 Hz.
(f) Compression was halted when a sharp drop appeared in the displacement-axial load curve. The upper platen was then raised, and the failed specimen was carefully removed, indicating that sliding failure of the Sn58Bi alloy plug had occurred.

The shear failure at the alloy plug/casing interface progresses through three distinct stages:

Compaction Stage (O–S_1_): During this phase, a relatively large displacement is observed as the alloy fills voids and micro-gaps formed during the initial plug forming.

Elastic Deformation Stage (S_1_–S_2_): Axial pressure rises rapidly while the alloy plug undergoes elastic deformation.

Sliding Stage (S_2_–S_3_): Relative shear displacement occurs at the interface between the alloy plug and the casing.

### 3.3. Gas-Tightness Tests of Alloy Plugs

**Figure 6:**
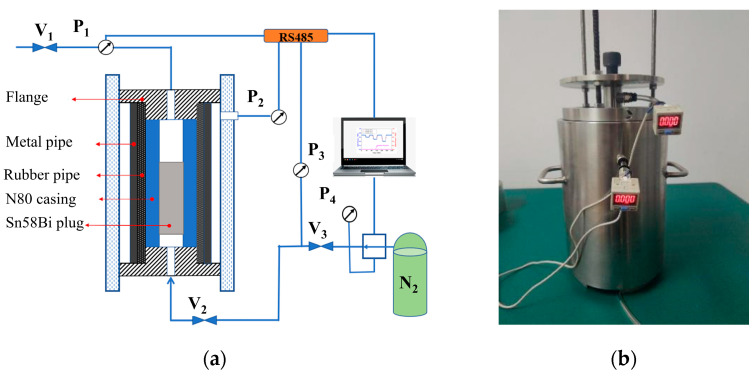
(**a**) Air Seal performance testing system and (**b**) physical test device of gas-tightness.

Gas-tightness is a critical performance criterion for assessing the sealing performance of LMPAs plugs. To investigate the gas sealing performance of Sn58Bi alloy plugs and the degree of gas-tightness deterioration following mechanically induced shear sliding, a gas-tightness testing system was designed and fabricated, as illustrated in Figure 6. This system comprises pneumatic valves V_1_, V_2_, and V_3_, along with pressure transducers P_1_, P_2_, P_3_, and P_4_. Pressure transducers P_1_ and P_2_ (range: 0–1 MPa, accuracy: 0.001 MPa) are connected in-line with the gas-tightness test assembly to record real-time pressure fluctuations at the upper outlet and the annular leakage path of the alloy plug. Transducers P_3_ and P_4_ (range: 0–25 MPa, accuracy: 0.1 MPa) are connected to the gas supply system and the inlet of the test assembly, respectively, to monitor the inlet pressure exerted on the lower surface of the alloy plug. High-purity nitrogen (N_2_) was used as the pressurizing medium, and the gas supply system was capable of delivering pressures up to 25 MPa.

The detailed experimental procedure is outlined as follows:

(a) Casing test specimens containing Sn58Bi alloy plugs, previously cured at 30 °C for 24 h, were removed and allowed to cool to room temperature (20 °C) and held at this temperature for 5 h.
(b) Each specimen was installed and sealed inside the gas-tightness test assembly.
(c) The nitrogen supply system was activated, and the outlet pressure was increased incrementally at a rate of 2 MPa/5 min by adjusting the supply valve.
(d) Nitrogen gas was introduced to the region below the alloy plug. A detectable pressure reading from transducer P_2_ (located above the alloy plug) was considered indicative of gas-tightness failure.
(e) Pressure data were continuously recorded using a data acquisition system, and the specimen was then removed from the test assembly.
(f) A separate set of specimens, prepared under conditions identical to those in step (a), were subjected to push-out shear tests using a universal testing machine. Steps b–e were repeated for these sheared specimens.
(g) The gas-tightness performance of the Sn58Bi alloy plugs before and after shear-induced sliding failure was compared to quantify the extent of performance deterioration.

### 3.4. Microstructural Analysis

Scanning electron microscopy (SEM) was utilized to characterize the microstructural features of various Sn-xBi alloys, thereby identifying a suitable SnBi alloy for oil casing sealing applications. Representative specimens (6 mm × 6 mm × 6 mm) extracted from the cast bulk material were used for systematic and statistical characterization of interfacial microstructures via field-emission scanning electron microscopy (FE-SEM, Quanta 650, Thermo Fisher Scientific, Hillsboro, OR, USA) at 15 kV. In addition, to assess the bonding integrity of the formed alloy plug surface and the alloy plug/casing interface, casing samples containing alloy plugs were sectioned perpendicular to the interface after gas-tightness testing. An optical microscope (Keyence VHX-5000, Osaka, Japan; detailed specifications are presented in Table 3) was used to examine the surface and cross-sectional gaps at the alloy–casing interface of samples tested under different overburden pressures. This microstructural analysis enabled a quantitative assessment of the effect of overburden pressure on the sealing integrity of the alloy plug/casing interface.

## 4. Results and Discussion

### 4.1. Ambient Temperature Effect on Ultimate Shear Strength of sn58bi Alloy Plugs

When Sn58Bi alloy plugs are employed for casing sealing applications, no chemical reactions or interfacial bonding occur at the alloy plug–casing interface. Consequently, the plugging tightness and integrity depend primarily on the radial expansion force generated during alloy solidification—which exerts a pressing force on the inner wall of the casing—and the resulting frictional resistance that enables mechanical anchoring. The mechanical properties of the Sn58Bi alloy are highly temperature-dependent: elevated temperatures induce a significant degradation in its mechanical performance. For LMPAs (e.g., Sn58Bi), elevated ambient temperatures tend to promote creep deformation, thereby leading to a reduction in the ultimate shear strength of the alloy plug-casing mechanical seal interface. The average shear strength of the Sn58Bi alloy plug can be mathematically expressed as follows [21]:

(1) $$\tau_{a}=\frac{\sigma_{z}r}{2L}$$

where *τ_a_* denotes the average shear strength (MPa) at the alloy plug/casing interface; *σ*_z_ represents the axial stress (MPa) at the end of the LMPA plug; *r* is the radius (mm) of the LMPA plug, and *L* refers to the length (mm) of the LMPA plug.

**Figure 7:**
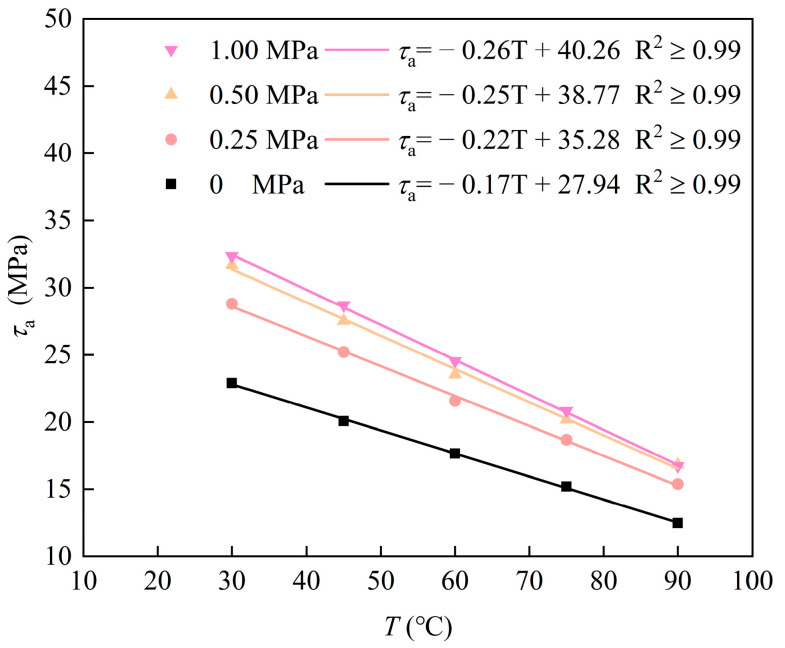
Effect of ambient temperature variation on the shear strength of Sn58Bi alloy plugs.

To investigate the effect of ambient temperature on the ultimate shear strength of Sn58Bi alloy plugs, push-out tests were conducted on specimens fabricated under four distinct overlying axial pressures. Varying the heating temperature of the constant-temperature oven and holding the specimens at each target temperature for more than 5 h allowed simulation of the shear strength resistance of the alloy plug under different ambient conditions. The results under different ambient temperatures are illustrated in Figure 7. To ensure the accuracy of the test data, the push-out tests were conducted on Sn58Bi alloy plugs with five different lengths (50, 75, 100, 125 and 150 mm) under each ambient temperature condition, and the average value was adopted for the test results.

Experimental results indicate that the ultimate shear strength of the alloy plug for zonal isolation decreases significantly with the increase in ambient temperature, which is attributed to creep and other related factors. This monotonic and gradual variation leads to an approximately linear relationship between shear strength and ambient temperature. Within the temperature range of 30 °C to 90 °C, the decrease in the ultimate shear strength of the alloy plug exhibits a linear correlation with the rise in ambient temperature. The linear decrease in shear strength mainly arises from the continuous and uniform thermal softening of the alloy matrix with increasing temperature. Specifically, for every 1 °C increase in ambient temperature, the ultimate shear strength of the alloy plug decreases by 0.225 MPa. Accordingly, the relationship between the maximum shear sliding stress *σ*_z_ at the interface of the Sn58Bi alloy plug and casing and the ambient temperature *T* can be expressed as follows:

(2) $$\sigma_{z}=\frac{2L\left(-0.225T+A\right)}{r}$$

where *σ_z_* represents the axial stress (MPa) at the end of the Sn58Bi alloy plug; *r* denotes the radius (mm) of the Sn58Bi alloy plug; *L* refers to the length (mm) of the Sn58Bi alloy plug; *T* is the ambient temperature (°C), with 90 °C ≥ *T* ≥ 30 °C; and A is a constant related to factors such as casing surface topography and the overlying axial pressure applied to the alloy plug (Quality of bonding between alloy plug and casing surface).

### 4.2. Effect of Overlying Axial Pressure on the Ultimate Shear Strength of Sn58bi Alloy Plugs

**Figure 8:**
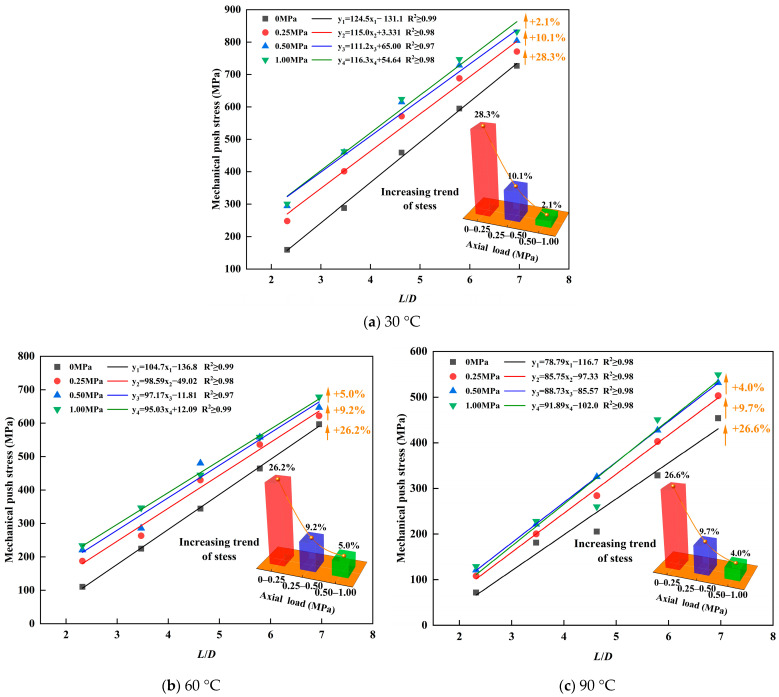
Results of push-out test under sealing and plugging of alloy plug formed under different overlying axial pressure.

The overlying axial pressure fulfills two primary functions: it drives the molten alloy into the microscopic irregularities of the inner casing surface, thereby enhancing the interfacial bond strength between the alloy plug and casing, and it elevates the radial expansion force of the Sn58Bi alloy plug during its solidification process. Push-out tests were performed to quantify the maximum axial stress required to initiate shear failure of Sn58Bi alloy plugs under three distinct ambient temperature conditions, with the experimental results illustrated in Figure 8. Experimental results demonstrate that, under different ambient temperatures, the pressure-bearing capacity of the alloy plug increases monotonically with elevated overlying axial pressure. A more than 26% improvement in mechanical push stress is observed as the overlying axial pressure rises from 0 MPa to 0.25 MPa for all tested temperatures. Nevertheless, the growth rate diminishes significantly with further pressurization, and the minimum increment in mechanical push stress is only 2.1% when the pressure increases from 0.5 MPa to 1 MPa. Accordingly, applying an overlying axial pressure of 0.5 MPa is sufficient to effectively strengthen the interfacial integrity between the alloy plug and casing and the radial expansion effect of the plug. However, when the overlying axial pressure exceeds 0.5 MPa, the incremental pressure primarily contributes to enhancing the radial expansion effect, with diminishing returns for further enhancement of the interfacial bond strength.

**Figure 9:**
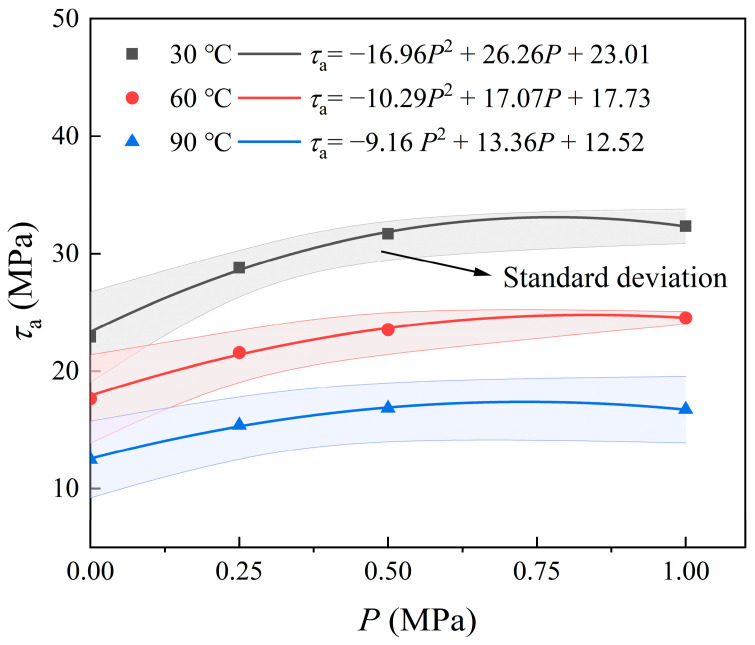
Overlying axial pressure effect on ultimate shear strength of Sn58Bi alloy plugs and casings.

For the same temperature conditions, the ultimate shear strength of the Sn58Bi alloy plug was determined for different plugging lengths (50, 75, 100, 125, and 150 mm). The mean values and corresponding standard deviations were calculated from the experimental data. As illustrated in Figure 9, the ultimate shear strength of the alloy plugs increases with the rise in overlying axial pressure, although the rate of increase gradually decreases. Specifically, the experimental results demonstrate that an initial forming axial pressure of up to 0.5 MPa can effectively improve the ultimate shear strength at the alloy plug/casing interface.

**Figure 10:**
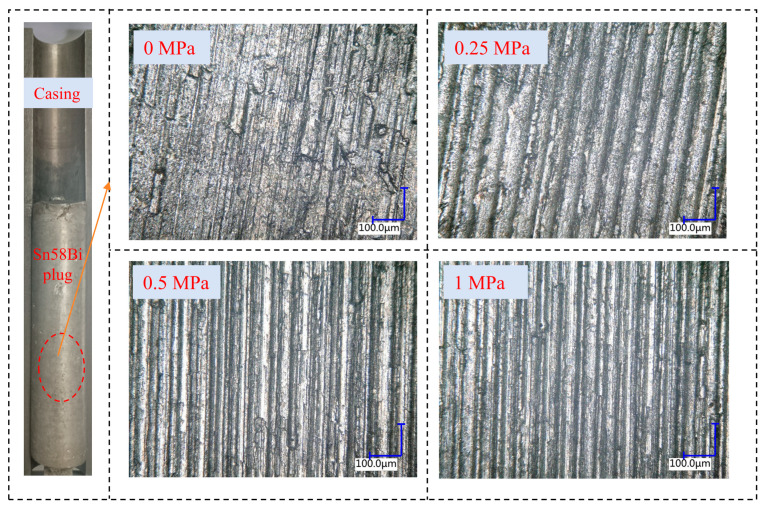
Microcosmic picture of Sn58Bi alloy plug surface with or without overlying axial pressure.

To further analyze the effect of overlying axial pressure on the integrity of the alloy plug, specimens from the plug formation tests were sectioned and examined via optical microscopy. The analysis focused on the plug surface and the alloy plug/casing interface, where the latter refers to the bonding interface between the alloy plug and casing. As illustrated in Figure 10, microscopic images of the plug surface were acquired by sectioning Sn58Bi alloy plug/casing samples with the same zonal isolation length but formed under different levels of overlying axial pressure. Comparisons of the micrographs from the central region of the plugs demonstrate that the interfacial bonding integrity of Sn58Bi alloy plugs formed under applied overlying axial pressure is significantly superior to that of plugs formed without any overlying axial pressure.

**Figure 11:**
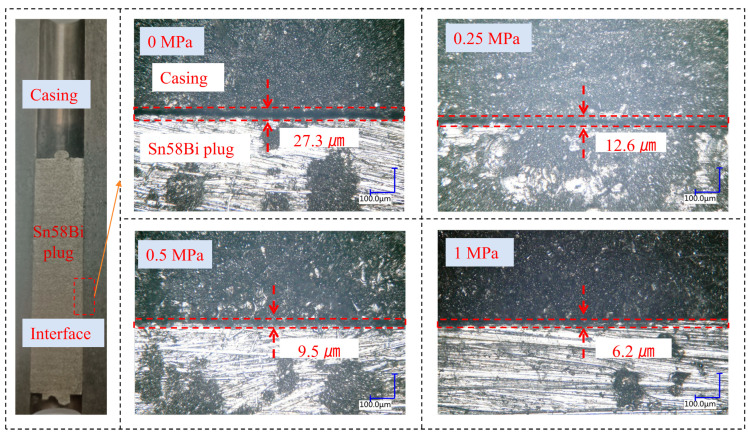
The interface gap of Sn58Bi alloy plug/casing changes with the overlying axial pressure.

Upon sectioning Sn58Bi alloy plug/casing specimens formed under different levels of overlying axial pressure, the distribution of interfacial gap sizes at the alloy plug/casing interface was observed via optical microscopy, as illustrated in Figure 11. The experimental results demonstrate that the application of overlying axial pressure significantly enhances the interfacial bonding integrity, thereby effectively reducing the size of interfacial gaps. When the overlying axial pressure reaches 0.5 MPa or higher, the contact between the alloy plug surface and the casing becomes nearly complete, with interfacial gaps of less than 10 µm.

### 4.3. Mechanical Plugging Failure Effect on Sealing Performance of sn58bi Alloy Plugs

**Figure 12:**
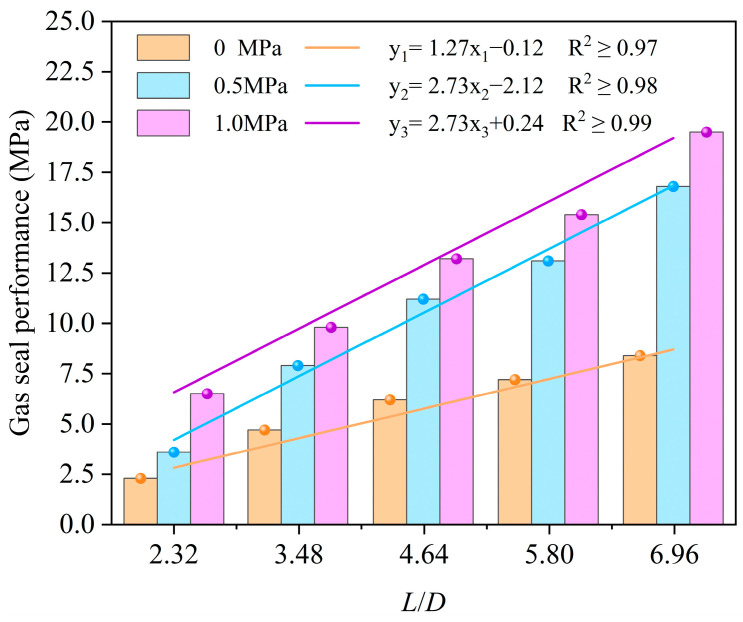
Gas-sealing performance of Sn58Bi alloy plugs.

To investigate the evolution of gas-tightness subsequent to the mechanical sealing failure of Sn58Bi alloy plugs (i.e., after the occurrence of relative shear displacement between the alloy and casing), the gas-tightness of Sn58Bi alloy plug/casing specimens was characterized prior to push-out tests for specimens with varying aspect ratios (*L*/*D*) and under graded overlying axial pressures. As presented in Figure 12, the gas-sealing performance of the alloy plugs exhibits a linear increase with an elevation in the *L/D*. Moreover, across the three examined levels of overlying axial pressure, the gas-sealing performance is further enhanced with an increase in the forming pressure.

**Figure 13:**
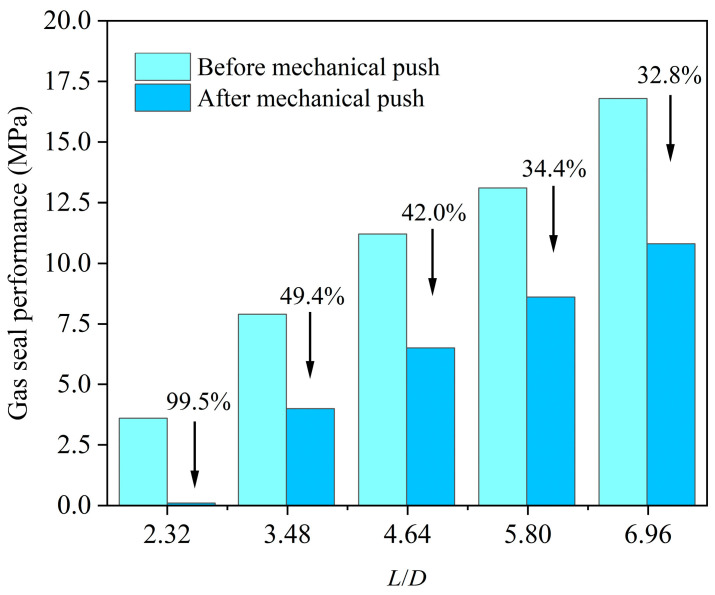
Gas-sealing performance of Sn58Bi alloy plug before and after push-out tests.

In Figure 12, the gas-tightness of the test group fabricated without overlying axial pressure exhibits only a marginal enhancement with an increase in *L/D*. This limited improvement is attributed to the inadequate structural integrity of the plugs during fabrication. To eliminate the potential bias induced by this factor, gas-tightness tests were conducted on specimens with five distinct *L/D*—all fabricated at an overlying axial pressure of 0.5 MPa—both before and after push-out testing. As illustrated in Figure 13, Sn58Bi alloy plugs having an *L/D* of 2.32 lose nearly all gas-tightness subsequent to mechanical shear failure. However, with an increase in *L/D*, the residual gas-tightness after shear failure increases progressively. For plugs having an *L/D* of 3.5 or higher, more than 50% of the original gas-tightness is retained even after the occurrence of mechanical shear displacement.

**Figure 14:**
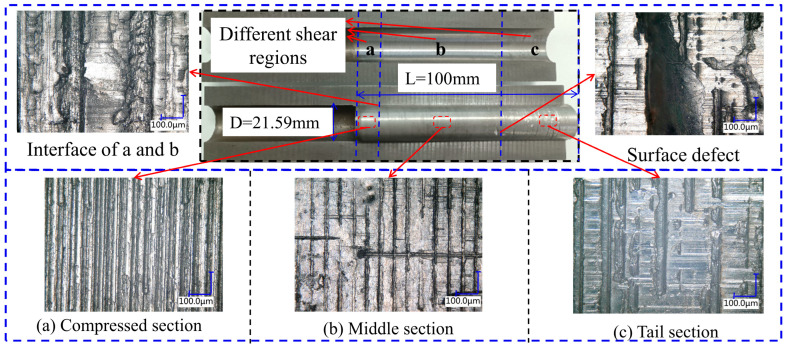
The microscopic surface of Sn58Bi alloy plug sample after the push-out tests.

After push-out testing, the specimens were sectioned, and the surfaces of the Sn58Bi alloy plugs were examined via optical microscopy to characterize the interface evolution following mechanical displacement. As presented in Figure 14, along the pushed section of the Sn58Bi alloy plugs, the surfaces after shear displacement correspond to Figure 14a–c in the top-to-bottom sequence. Figure 14a demonstrates that although the Sn58Bi alloy plug was displaced, the sealing interface between the alloy and the casing remained largely intact, indicating minimal interface damage at this location. In contrast, from Figure 14b to Figure 14c, the deterioration of the alloy plug/casing sealing interface becomes increasingly prominent, with visible gaps and detachment emerging as the distance from the top of the pushed section increases. This can be attributed to the fact that the alloy plug experiences global shear failure upon being pushed. The pressurized end Figure 14a is subjected to a high axial load, where the local alloy undergoes permanent plastic deformation and fills the annular gap between the plug and the casing, thereby retaining satisfactory sealing integrity. In contrast, the driving force is substantially attenuated when transferred to the bottom end Figure 14c, which fails to induce sufficient deformation in the alloy, leading to a significant reduction in sealing integrity. Under downhole high-pressure differential environments, once sliding-induced shear failure occurs at the Sn58Bi alloy plug/casing interface, the risk of seal failure increases along the pushed section in the order of c > b > a.

## 5. Conclusions

The feasibility of Sn58Bi LMPA as a casing plugging material was evaluated through simulation experiments, and the failure modes related to mechanical sliding-induced shear failure of the alloy plugs were analyzed. The experimental results confirm the potential of Sn58Bi alloy as an alternative casing sealing material, providing critical data to support the development of more reliable downhole plugging technologies.

The main conclusions drawn from this study are as follows:

(1) Ambient temperature exerts a significant influence on the performance of Sn58Bi alloy plugs. With increasing ambient temperature, the interfacial bond strength between the Sn58Bi alloy and casing decreases, resulting in a corresponding reduction in the ultimate shear strength of the plugs. Within the temperature range from 30 °C to 90 °C, this reduction exhibits a linear correlation with temperature elevation, with the ultimate shear strength decreasing by approximately 0.225 MPa per 1 °C increase in ambient temperature.
(2) The application of overlying axial pressure (0–1 MPa) during the solidification of Sn58Bi alloy plugs enhances the interfacial bonding quality between the alloy plug and casing, thereby improving both the mechanical bearing capacity and gas-tightness of the plugs. Overlying axial pressure below 0.5 MPa effectively improves interfacial bonding quality and promotes radial expansion of the plugs. In contrast, when the forming pressure exceeds 0.5 MPa, the additional axial force primarily contributes to enhanced radial expansion, with diminishing returns in further improving interfacial bonding performance.
(3) Shear failure occurs at the interface between Sn58Bi alloy plugs and the inner wall of the casing when the plugs are subjected to push-out displacement. Plugs with an *L/D* of 2.32 lose nearly all their gas-tightness following mechanical shear failure. As the *L/D* increases, the residual gas-tightness of the plugs after shear failure improves progressively. For plugs with an *L/D* of 3.5 or higher, more than 50% of the original gas-tightness is retained even after the occurrence of mechanical shear displacement, indicating good residual sealing performance.

## Figures and Tables

**Table 1:** Properties of Sn58Bi alloys [8,9].

Properties	Melting Point (°C)	Surface Tension at 155 °C (mN/m)	Thermal Expansion Coefficient × (°C^−1^)	Volume Change (from Liquid to Solid)	Density at 21 °C (g/cm^3^)	Elastic Modulus at 21 °C (GPa)
Numerical	138	438	1.5 × 10^−7^	+0.77%	8.72	47.2

**Table 2:** Properties of N80 carbon-manganese steel [16,17].

Properties	Thermal Expansion Coefficient × (°C^−1^)	Density (g/cm^3^)	Elastic Modulus at 30 °C (GPa)	Elastic Modulus at 60 °C (GPa)	Elastic Modulus at 90 °C (GPa)
Numerical	1.3 × 10^−5^	7.85	196	169	158

**Table 3:** Specifications of the VHX-5000 optical microscope employed.

Image Sensor	Virtual Pixels	Frame Rate	Magnification
1/1.8 inch	1600 (H) × 1200 (V)	0–50 fps	0–100

## Data Availability

The original contributions presented in the study are included in the article, further inquiries can be directed to the corresponding author.

## Abbreviations

The following abbreviations are used in this manuscript:

LMPA	Low-melting-point alloy
CCUS	Carbon Capture, Utilization, and Storage
P&A	Plugging and Abandonment
LMPAs	Low-melting-point alloys
BiSn	Bismuth-tin
SEM	Scanning electron microscopy
Bi	Bismuth
Sn	Tin
ID	Inner diameter
OD	Outer diameter
*L/D*	Aspect ratio
